# Enhancing online interaction through avatar-based dialogue systems utilizing the approaching movement

**DOI:** 10.1371/journal.pone.0327712

**Published:** 2025-07-18

**Authors:** Ayaka Ueda, Hamed Mahzoon, Kazuki Sakai, Hiroshi Ishiguro, Yuichiro Yoshikawa

**Affiliations:** 1 Graduate School of Engineering Science, The University of Osaka, Toyonaka, Osaka, Japan; 2 Institute for Open and Transdisciplinary Research Initiatives (OTRI), The University of Osaka, Suita, Osaka, Japan; Tongji University, CHINA

## Abstract

The rise in online interactions has introduced multiple challenges, including confusion during virtual meetings and fatigue associated with prolonged video conferencing. To address these issues, this study advocates using computer graphics (CG) avatars in dialogue systems that do not rely on camera feeds. Avatars have the potential to diminish gaze-related misunderstandings and reduce dissatisfaction arising from viewing oneself in video calls. Moreover, fatigue from continuous face-to-face interaction in multi-person conversations can be mitigated by avatar usage. Previous studies indicate that avatars’ verbal and nonverbal communication play significant roles in facilitating social support and cooperation in online environments. In the field of robotics, nonverbal cues including movement and spatial positioning are crucial for improved communication. For instance, robotic movement can indicate shifts in attention and speaker preferences during human–robot interactions. However, the impact of movement in dialogue contexts, especially in comparison to a neutral stance lacks thorough evaluation. This study conducted a video-based experiment to assess the impact of a robot’s approach movement on the perception of emotions and intentions in a multi-robot interaction scenario. The results suggest that approaching movement can enhance the expression of intentions and emotions, indicating desired turn-taking and conveying perceived impressions of a robot as positive or negative. Future research will focus on implementing these findings in real-time conversational experiments.

## Introduction

In recent years, there has been a surge in online interactions, presenting both opportunities and challenges. Confusion in online meetings due to the difficulty in understanding the direction of gaze of the participants has been well documented [1–4]. Numerous attempts in online conferencing systems have aimed to address issues related to gaze and face orientation [5,6].

Concurrently, the extended duration of video calls has been identified as a source of fatigue [7–10], with specific concerns on self-view during video calls exacerbating appearance dissatisfaction [8,9]. Additionally, fatigue from constant attention in multi-person conversations contrast sharply with the dynamics of in-person conversations [10].

In response to these challenges, employing computer graphics (CG) within dialogue systems emerges as a viable, non-camera-based alternative. Research by Takano *et al*. highlighted the impact of avatars’ verbal and nonverbal communication in facilitating social support [11]. Evidence from massively multiplayer online (MMO) games demonstrate the role of nonverbal cues in fostering cooperation among players [12].

In the field of robotics, the exploration of nonverbal communication has been aimed at facilitating smoother and more nuanced interactions. Nonverbal communication modalities such as facial expressions [13–16], gestures [17,18], and colors [19–21], have been researched to express mental states such as robot intentions. Particularly, movement and positioning are known for their ability to express intimacy in human communication [22] and O-space, a space indicating participation in a conversation [23]. These areas of nonverbal communication are garnering attention in robot–human interaction [5,24,25]. For example, Kuzuoka *et al*. demonstrated the effectiveness of utilizing body rotation and the direction of the head and eyes to communicate the robot’s intention to “alter the direction of the person’s attention” [24]. Studies on robot interactions have investigated how robots can express emotions and intentions by moving closer or away in scenarios involving three robots [26].

Assessing the effect of approaching movement within the flow of the dialogue or in comparison to a neutral state such as no movement, has remained a challenge. Therefore, this study conducted a video viewing experiment aimed at the progression of multi-person dialogue by improving the avatar communication of emotions and intentions. This experiment focused on evaluating observer perception of robot approaching movement when three robots interact. Specific attention was given to the approaching movement of one robot approaching another, which is thought to contribute to the speaker identification and predicting the next speaker.

## Materials and methods

### Robotic virtual conversation environment

**Fig 1:**
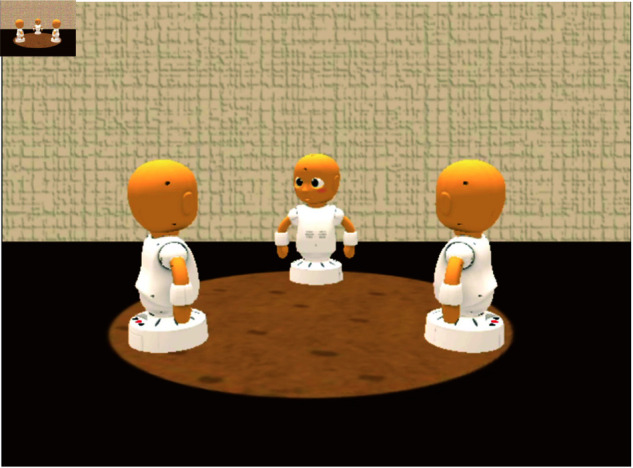
Robotic virtual conversation environment.

In this experiment, we used a virtual communication environment accessible via a web browser, featuring three CG robot avatars facing each other (see Fig 1). Users may control these robots to gesture, speak, or move to any arbitrary position within the room.

### Parameters for approaching movement in conversation

To simplify the study, we defined the speaking and moving robots and the direction of approaching movement of the active robot within the conversation among the three robots. We conceptualized a simple conversation among the three robots, divided into two distinct scenes. In the first scene, one of the three robots was designated to speak. Following this, in the second scene, the next instance of speech may be by the same or a different robot. In addition to the approaching movement parameter, the speaking robots in the experimental videos also exhibited other nonverbal behaviors to simulate a natural conversational setting. These included predefined arm gestures and head movements accompanying speech, as well as specific gaze directions. These co-occurring nonverbal cues were consistently applied across all video conditions (both with and without approaching movement) based on a set of standardized criteria, aiming to provide a realistic interaction context while focusing on the effect of the approaching movement itself.

Regarding the timing of the approaching movement, we focused on ‘movement as a reaction to the initial speech’. Based on our assumption that a natural reaction to the first speaker’s utterance would occur around the beginning of the subsequent speaker’s turn, we set the timing of the approaching movement to coincide with the start of the second robot’s speech. The experimental videos were automatically generated using Javascript, which ensured that the approaching movement consistently began precisely at the onset of the second speaker’s utterance across all conditions involving movement. Therefore, in this study, the timing of the approaching movement was fixed at this specific point and was not treated as a variable parameter for analysis. Investigation into the effects of varying movement timing, such as delays or reactions at different points in the conversation, is considered important but is beyond the scope of this particular experiment.Regarding the approaching distance, our aim in this study was to investigate the effect of the act of approaching itself within a conversational context, rather than manipulating specific distances or analyzing effects related to Hall’s proxemics categories [22]. Therefore, approaching distance itself was not treated as a variable parameter for analysis in this experiment.

Here, only the robot at the center is programmed to move. As detailed in Table 1, the experimental design incorporated nine different speaking patterns, in which three patterns were possible for both the first and second speakers. The direction of approaching movement was set as either an approach toward the robot on the right, or to remain static. The direction of approaching movement was set as an approach specifically toward the robot on the right side. This design choice was made primarily for simplicity in constructing the experimental conditions, based on an initial assumption that perceptual effects related to movement direction would be symmetrical between left and right.

**Table 1:** Experimental patterns for sequence of speech.

	First Speaker	Second Speaker
1	Left	Left
2	Left	Center
3	Left	Right
4	Center	Left
5	Center	Center
6	Center	Right
7	Right	Left
8	Right	Center
9	Right	Right

These evaluations have been discussed in the Experiment section.

### Ethics statement

All the participants agreed with the written consent from approved by the ethics committee for research involving human subjects at the Graduate School of Engineering Science, Osaka University (approval number R1-7-2). The experiment was conducted from 2021/4/21 to 2021/5/4. All relevant data are within the manuscript and its Supporting Information files.

### Experiment

#### Hypothesis.

In order to investigate whether the approaching movement, occurring at a specific timing (i.e., the beginning of the second speaker’s utterance), influences the subjective evaluation of the emotions and intentions of the moving robot, based on the sequence of the robots’ speaking, we conducted a video watching experiment facilitated through crowdsourcing.

#### Measures.

**Fig 2:**
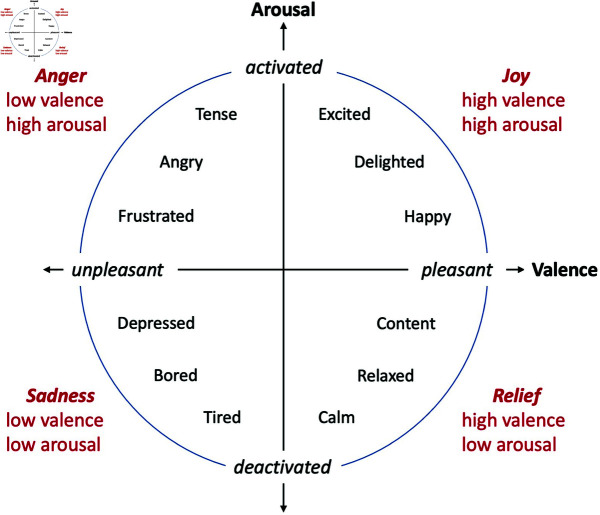
The circumplex model of affect with two axes of valence and arousal [27].

The measures for this study were the subjective evaluations of participants regarding their perception of the robot at the center, including the aspects of emotion, intention, animacy, sociability, agreement or disagreement with the robots on either side, positive or negative impressions, and predictions on who would speak next. To assess the robot’s emotion, we used Russell’s circumplex model of affect, which involves questioning participants on the perceived valence (pleasure or displeasure) and arousal (high or low) across the two axes [27] (see Fig 2). On the questionnaire, an image of Russell’s Emotion Periphery Model was presented and the following statement was made: “If emotions are classified according to whether they are active or not, pleasant or unpleasant, they can be classified as shown in the figure. Animacy was measured using Bartneck Godspeed animacy scale [28]. Specifically, we asked “Please rate your impression of the robot on these scales: Dead/Alive, Stagnant/Lively, Mechanical/Organic, Artificial/Lifelike, Inert/Interactive, Apathetic/Responsive” and rated the sum of the five-point ratings for each. The sociality of the robot was measured using a modified version of the KiSS-18 items that inquired about the perceived sociality of the robot rather than the participant’s own [29]. In particular, the following in Table 2 were used to evaluate the sum of a five-point rating for each of the following. We conducted a subjective evaluation using a structured questionnaire.

**Table 2:** Question items for evaluating sociality of the robot [29]. The text of the table is the translation from original Japnese questionnarie.

	Question item
1	When talking with others, it tends not to have many interruptions in the conversation.
2	It can effectively instruct others on what it wants them to do.
3	It can skillfully help others.
4	When someone is angry, it can calm them down well.
5	It can quickly start a conversation even with strangers.
6	Even if trouble arises with people around it, it can handle the situation skillfully.
7	When it feels scared or terrified, it can manage those feelings well.
8	It can skillfully reconcile with someone with whom there has been an awkward situation.
9	When working, it can decide what needs to be done and how to do it.
10	It can casually join in when others are talking.
11	Even when criticized by others, it can handle it well.
12	In its work, it can quickly find where problems lie.
13	It can frankly express its own emotions and feelings.
14	Even when receiving contradictory information from various sources, it can handle it well.
15	It can skillfully introduce itself to people it meets for the first time.
16	When it makes a mistake, it can apologize promptly.
17	Even if the people around it have different ideas from its own, it can get along well with them.
18	It doesn’t find it very difficult to set work goals.

#### Test.

A three-way ANOVA was conducted with three independent variables (IVs) and twelve dependent variables (DVs), to study the effect of using “Approaching Movement,” “First Speaker,” and “Second Speaker” as three between-subjects variables on the DVs. As timing of the approaching movement was a fixed parameter in this study (set at the beginning of the second speaker’s utterance) and not included as an independent variable in the ANOVA, the statistical analysis focused on the effects of Approaching Movement, First Speaker, and Second Speaker. Similarly, as approaching distance was a fixed change within the ‘approaching movement’ condition and not treated as an independent variable, the results do not include analysis specifically on the effects of varying approaching distance. DVs represented improvements in subjective evaluations, calculated by the difference between the evaluations of two videos, one without approaching movement and the other with stimuli (either no movement or with approaching movement). To simplify discussion, we focused exclusively on items related to the movement. For the same reason, the statistical report on non-significant effects was omitted.

#### Procedure.

First, the subjects watched a video of a stationary robot and answered the questionnaire. Following this, they were shown another video, either of the same stationary robot or an approaching movement toward the robot on the right, after which they again answered the questionnaire. In order to conduct the experiment in a between-subjects comparison, each subject was exposed to only two distinct videos as described above.

#### Subjects and apparatus.

The target group was designed to achieve an equal number of male and female subjects distributed within the age range of 20 and 59 years. Based on the priori power analysis, with effect size *f* = 0.2, significance level α=0.05, and statistical power 1−β=0.80, it was determined that 14 subjects per condition were required. As a result, 191 human subjects with a mean age of 40.87 (SD = 10.70) participated in this study, comprising 86 males and 105 females.

### Result

**Fig 3:**
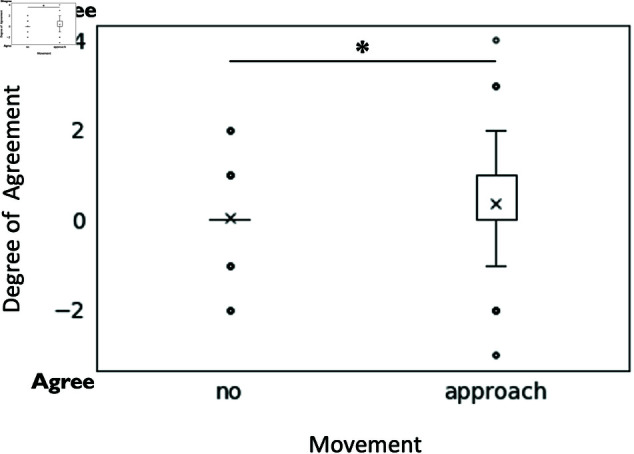
The effect of approaching movement on the robot’s expression of feelings toward the left robot. In the figure, the subject’s perception of the center robot’s agreement or disagreement with the left robot’s statements and opinions is shown.

For question one, “How do you feel the robot in the center feels about the statements and opinions of the robot on the left?”, we found a main effect of approaching movement (F(1, 190) = 4.25, p = .041, $partial\;\eta^{2}$ = 0.0234). Approaching movement (M = 0.37, SD = 1.21) perceived as significantly more oppositional toward the robot on the left, compared to without approaching movement (M = 0.04, SD = 0.71) (Fig 3).

**Fig 4:**
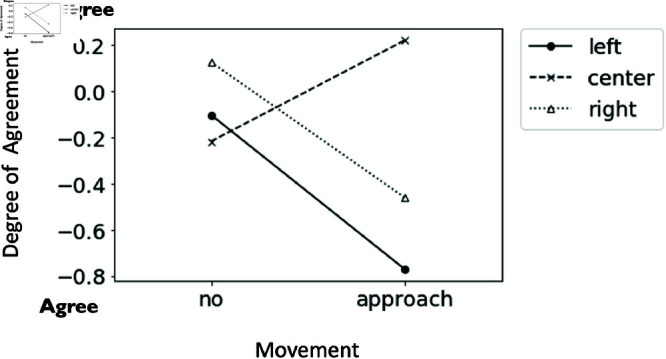
The interaction effect of approaching movement and the second speaker on the robot’s expression of feelings toward the right robot. The graph shows the extent to which the subjects perceived the center robot’s agreement or disagreement with the right robot’s statements and opinions.

In question two, “How do you feel the robot in the center feels about the statements and opinions of the robot on the right?”,a significant two-way interaction between approaching movement and the second speaker was observed (F(2, 190) = 6.90, p = .001, $partial\;\eta^{2}$ = 0.074). Simple main effects were identified when the second speaker was positioned on the left, center, and right (F(1, 63) = 5.38, p = .022, $partial\;\eta^{2}$ = 0.030; F(1, 63) = 3.95, p = .049, $partial\;\eta^{2}$ = 0.022; F(1, 62) = 7.38, p = .007, $partial\;\eta^{2}$ = 0.041). The results showed that when the second speaker was either the left or right robot, the moving robot (M = −0.77, SD = 1.09; M = −0.46, SD = 1.43) was significantly more agreeable to the right than the stationary robot (M = −0.10, SD = 0.49; M = 0.13, SD = 0.61). Conversely when the second speaker was the center robot, the moving robot (M = 0.22, SD = 1.26) was significantly more disagreeable to the robot on the right than the stationary robot without approaching movement (M = −0.22, SD = 1.01) (see Fig 4).

**Fig 5:**
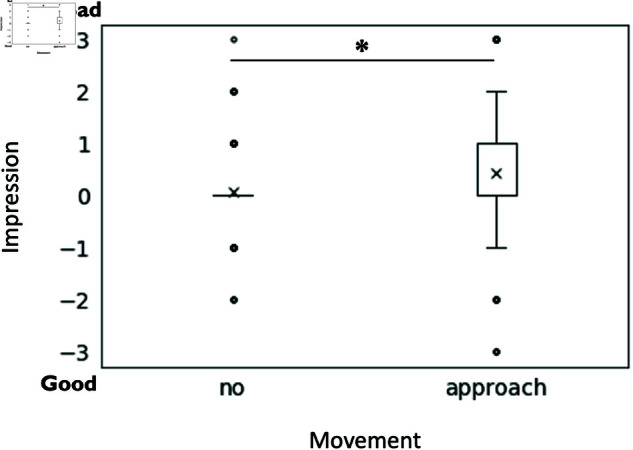
The effect of approaching movement on the expression of the robot’s impression about the left robot. The graph shows the extent to which the subjects perceived the center robot’s ‘good’ or ‘bad’ impression of the left robot.

Question 3, “What impression do you feel the center robot has of the robot on the left?” also showed a significant main effect of approaching movement (F(1, 190) = 4.27, p = .040, $partial\;\eta^{2}$ = 0.024). Approaching movement (M = 0.42, SD = 1.31) represented a significantly worse impression of the robot on the left compared to no movement (M = 0.07, SD = 0.83) (see Fig 5).

**Fig 6:**
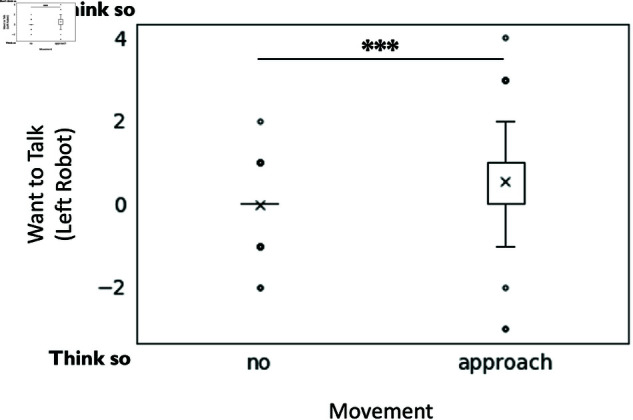
The effect of approaching movement on the expression of the center robot’s desire for the left robot to talk. The figure illustrates the subjects’ perception of the center robot’s desire, specifically whether they felt the center robot wanted the left robot to speak.

However for question four, “What impression do you feel the center robot has of the robot on the right?”, no significant effects of approaching movement on impressions were found. For question five, the question “To what extent do you feel that the robot in the center wants the robot on the left to talk?” a significant main effect of approaching movement (F(1, 190) = 13.8, p < .001, $partial\;\eta^{2}$ = 0.074), was found, indicating that the moving robot (M = 0.53, SD = 1 .17) was significantly more likely (M = −0.04, SD = 0.61) to be perceived as not wanting the robot on the left to speak, compared to in the absence of approaching movement (see Fig 6).

**Fig 7:**
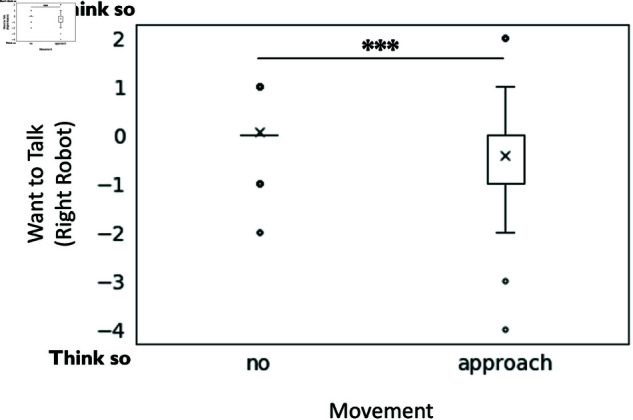
The effect of approaching movement on the expression of the center robot’s desire for the right robot to talk. The graph illustrates the subjects’ perception of the center robot’s desire toward the right robot, specifically whether they felt the center robot wanted the right robot to speak.

For question six, “To what extent do you feel that the robot in the center wants the robot on the right to talk?”, there was also a significant main effect of approaching movement F(1, 190) = 13.0, p < .001, $partial\;\eta^{2}$ = 0.070), indicating that the moving robot (M = −0.41, SD = 1.06) was significantly more likely to want the robot on the right to speak than the stationary robot (M = 0.06, SD = 0.61) (see Fig 7).

**Fig 8:**
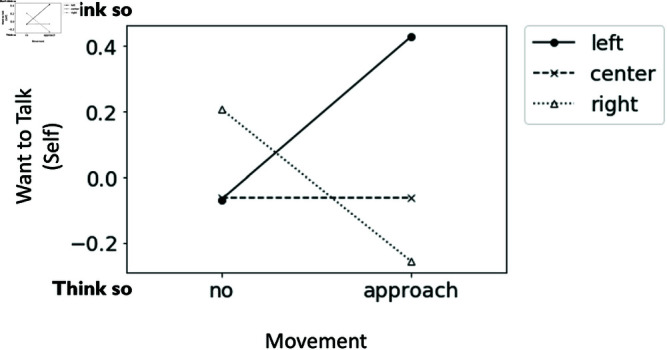
The interaction effect of approaching movement and the second speaker on the center robot’s desire to talk. The graph illustrates the subjects’ perception of whether the center robot appeared to want to talk.

Question seven, “To what extent do you feel that the robot in the center wants to talk?”, demonstrated a two-way interaction between approaching movement and the second speaker (F(2, 190) = 3.98, p = .021, $partial\;\eta^{2}$ = 0.044). A simple main effect was found when the second speaker was the robot on the right (F(1, 62) = 4.18, p = .043, $partial\;\eta^{2}$ = 0.024), and the moving robot (M = −0.08, SD = 0.86) was significantly more likely than the stationary robot (M = 0.07, SD = 0.92) to indicate that it wanted to speak (see Fig 8).

**Fig 9:**
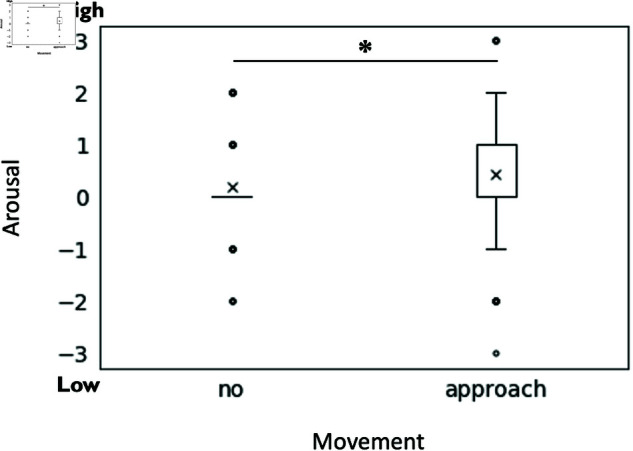
The effect of the robot’s approaching movement on the expression of arousal. The figure illustrates the main effect of approaching movement on the subjects’ perception of the center robot’s emotional arousal.

No significant results were obtained for question eight, “To what extent do you feel that the robot in the center wants to participate in the conversation?”. For question nine, “How low or high arousal do you feel about the emotions of the center robot to be?” we found a significant main effect of approaching movement (F(1, 190) = 3.95, p = .049, $partial\;\eta^{2}$ = 0.022), with approaching movement (M = 0.44, SD = 1.17)representing significantly higher activity and emotions than without approaching movement (M = 0.18, SD = 0.86) (see Fig 9).

For question ten, “How unpleasnt or pleasant do you feel about the emotions of the center robot?”, there were no significant results for movement. For question 11, i.e. the sociability of the robots, no significant effect was obtained.

**Fig 10:**
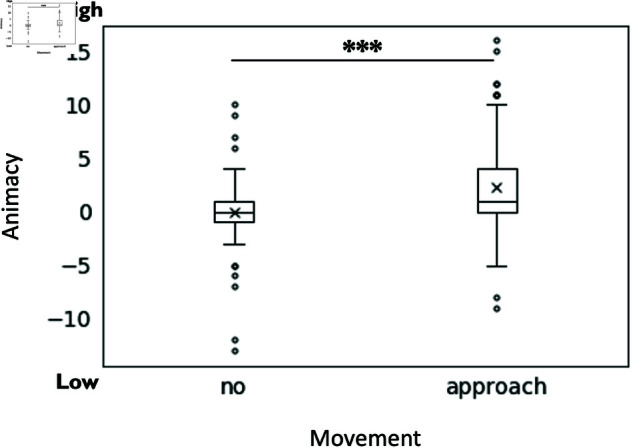
The effect of the robot’s approaching movement on its perceived degree of animacy. The graph shows that the center robot’s approaching movement significantly increased the perceived animacy.

Regarding the animacy of the robots in question 12, a significant main effect of approaching movement (F(1, 190) = 14.8, p < .001, $partial\;\eta^{2}$ = 0.079) was found, indicating that moving robots (M = 2.29, SD = 4.46) were perceived as significantly more animate than those without approaching movement (M = 0.00, SD = 3.27) (see Fig 10).

### Discussion

The results show that the expression of emotions and intentions can be enhanced by using approaching movement to get closer to other robots. This impact can vary with the sequence of speakers, depending on the speaker before the approaching movement, and the subsequent one.

We have discussed these results in terms of the intentions and emotions of the moving robot, and its intentions and impressions toward the robot it approaches (i.e. the robot on the right side in this experiment), and the robot it does not approach (i.e. the robot on the left side in this experiment).

#### Intentions and emotions of moving robot.

The study revealed that the moving robot could express high arousal, emotion, and animacy by approaching movement. In addition, it was found that the moving robot could express desire to converse when the robot being approached was the second speaker.

However, no specific effect on the degree of the valence of the robot was revealed in this study. This suggests that while approaching movement can represent a robot’s high level of arousal, animacy, and positive attitude toward conversation, the degree of valence, i.e. pleasant or displeasure, depends more on the content of the conversation than motion.

#### Intentions and impressions of moving robot on approached robot.

The results indicate that the moving robot could express its desire for the approached robot to speak, through its approach. Moreover, the moving robot could express disagreement with the approached robot when the second speaker was the moving robot, and agreement when the second speaker was either the approached or non-approached robot. The variation in approval or disapproval based on the identity of the second speaker, while employing the same movement, suggests that the speaker at the time of approaching movement is important in avatar dialogue. However, the study did not yield significant results for impressions about the approached robot. This suggests that the expression of impressions on the approached robot is more influenced by the content of the conversation rather than physical action.

#### Intentions and impressions of moving robot about non-approached robot.

The study discovered that the moving robot could express disagreement and a negative impression of the robot it did not approach. Additionally, it was observed that the moving robot could not express lack of desire to communicate with the non-approached robot through approaching movement. The results suggest that approaching movement may convey disengagement or disapproval of the non-approached robot. Recognizing this aspect is crucial in preventing unintended communication of such sentiments in actual dialogues.

#### Limitations and future work.

While this study did not manipulate specific distance categories or analyze the effects of crossing boundaries between them, the act of moving closer itself appears to shift the perceived social dynamics and convey a stronger intention to engage. This suggests that the spatial dimension, particularly changes in distance through movement, plays a significant role in nonverbal communication in avatar-based interactions, resonating with Hall’s concept that different distance zones are associated with varying degrees of social intimacy and communication styles. Building upon this, it is plausible that moving away could signal a decreased intention to participate or even withdrawal from the conversation, as indicated by the inverse relationship suggested by our results on approaching. Further research is needed to systematically investigate the effects of varying approaching distances, exploring their relationship with Hall’s categories, as well as the communicative functions of moving away in conversational contexts.

A notable limitation of the current study is the exclusive use of approaching movement directed towards the robot on the right side. This design was based on the simplifying assumption of symmetrical perceptual effects for movement towards the left. However, this might represent a biased assumption, as the direction of movement could potentially influence perception in asymmetric ways due to factors such as cultural reading habits or inherent spatial biases in human perception. Furthermore, the initial position of the moving robot within the group (e.g., being in the center) might also interact with the direction of movement to affect how the action is perceived. Therefore, future research is needed to investigate the effects of approaching movement towards the left side and to explore how the moving robot’s position within the group influences the interpretation of its nonverbal cues. Addressing these aspects will contribute to a more comprehensive understanding of the role of spatial dynamics in multi-party avatar-based communication.

Another limitation of this study concerns the presentation of the approaching movement concurrently with other nonverbal behaviors such as arm gestures and head movements. While these co-occurring cues were standardized across conditions, their presence means that the observed effects may be due to the pure approaching movement, or potentially the result of interactions between the approaching movement and these other nonverbal cues. For instance, specific gestures might amplify or modulate the perceived meaning of approaching. Evaluating the precise nature of such interactions and their potential bias implications on the interpretation of approaching movement’s effect was beyond the scope of this experiment. Therefore, future research should systematically investigate the interaction effects between approaching movement and other nonverbal cues, exploring how different combinations of movement, gestures, and gaze influence the perception of avatar communication.

Furthermore, while this study focused solely on approaching movements, future investigations should systematically explore the impact of diverse movement patterns, including retreating and lateral movements. We are confident that deeper insights can be gained by examining the effects of these movements in various conversational scenarios (e.g., decision-making, turn-taking, agreement, disagreement). To assess how well robot nonverbal cues align with natural human communication, human comparison studies are essential. We plan experiments where participants will compare their impressions of both humans and robots exhibiting the same movements in similar conversational contexts, which will allow us to objectively evaluate the naturalness and acceptability of robot nonverbal expressions. The current reliance on subjective measures also presents a limitation, and we acknowledge that controlling dialogue content more rigorously in future work could help to disambiguate the interplay between verbal and nonverbal effects. Therefore, real-time experiments are crucial for evaluating the practical effectiveness of our findings. Beyond simply measuring the impression conveyed by movement, we intend to measure its impact on the actual flow of dialogue (e.g., whether participants agree with the robot, the smoothness of the conversation). This will involve integrating objective metrics like eye-tracking to analyze participant attention and emotional states in greater detail. Should we be able to create an environment where participants can control their own avatars, we will also observe their avatar positioning after another avatar approaches. By observing these interactive reactions, we believe we can multi-dimensionally evaluate the practical effectiveness of nonverbal cues, while also acknowledging the practical challenges associated with implementing such real-time, interactive experiments.

### Conclusion

In this paper, we discussed a video watching experiment to investigate how a robot’s approaching movement and the sequence of speaking influences subjective perceptions of the robot’s emotions and intentions. Statistical analysis of data collected from the subjects revealed that certain emotions and intentions that can be more effectively expressed for the moving, the approached, and the non-approached robots. Furthermore, these expressions differ depending on the speaker at the time of approaching movement.

Incorporating such approaching movements into conversations allows for a more nuanced expression of intentions, including preferences among multiple speakers and the avoidance of certain topics. We believe that this kind of approaching movement can be applied to have intended turn-taking, such as expressing who you want to talk to or what you do not want to talk about. By combining approaching movement with facial expressions and gestures, emotional expression may be substantially improved. Furthermore, approaching movement may be used as a tool to express favorable or unfavorable impressions toward a particular robot.

To fully realize the potential of these findings and address the acknowledged limitations, the next phase of this research would involve conducting real-time conversational experiments using a system that incorporates these movement dynamics to further understand and optimize nonverbal communication in robotic interactions. The next phase of this research would involve conducting real-time conversational experiments using a system that incorporates these movement dynamics to further understand and optimize nonverbal communication in robotic interactions. Specifically, we intend to measure participant gaze patterns to objectively assess their attention and areas of focus during interactions. If participants are able to control their avatars, we will also closely observe their avatar positioning following another avatar’s approach. This will provide valuable insights into how participants react to and position themselves in response to the robot’s nonverbal cues, offering a more robust understanding of the real-world impact of our findings.

## Supporting information

S1 File Rawdata.zip Rawdata of the experiment. (ZIP)

S2 File questionnairy.pdf Questionnairy used for the experiment. (PDF)

S3 File 01.mp4 an example video (the robot remaining static). (MP4)

S4 File 02.mp4 an example video (the robot approaching toward the right robot). (MP4)
