# Supplementary material for: Enhancing online interaction through avatar-based dialogue systems utilizing the approaching movement
Source: PLoS One. 2025 Jul 18;20(7):e0327712. doi: 10.1371/journal.pone.0327712 (PMC12273998; doi:10.1371/journal.pone.0327712)
Supplement: S2 File — (PDF) [file pone.0327712.s002.pdf]

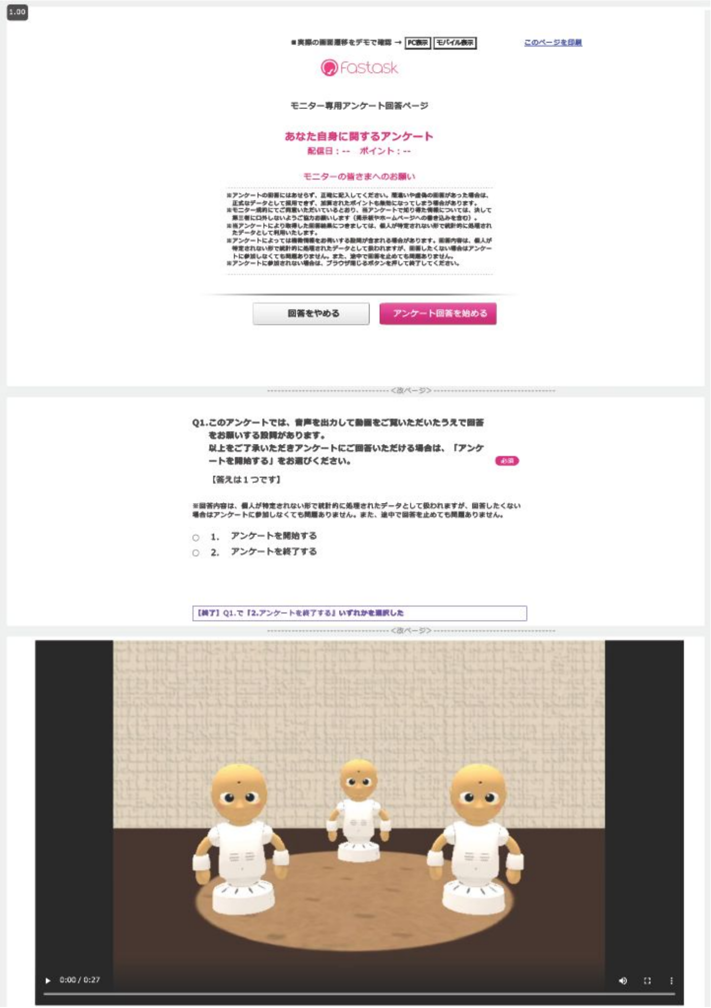

Survey About Yourself

To All Monitors

- Please answer the survey calmly and accurately. If there are any mistakes or false answers, they may not be adopted as valid data, and any awarded points may become invalid.
- As agreed in the monitor terms, please do not disclose any information obtained through this survey to third parties (including posts on bulletin boards or websites).
- The responses obtained from this survey will be statistically processed in a form that does not identify individuals.
- Some surveys may include questions about sensitive information. Responses will be statistically processed in an anonymous format. If you do not wish to answer, you may refrain from participating. You may also stop answering at any time.
- If you choose not to participate, please click the close button on your browser to exit.

------------------------- Page Break -------------------------

Q1. This survey includes questions that require you to watch videos with audio before answering. If you agree to this and would like to participate, please select "Start the survey."

[Choose only one answer]

- The responses will be processed statistically and anonymously. If you do not wish to answer, there is no problem with choosing not to participate or stopping midway.

1. Start the survey
2. Exit the survey

[End] If you selected "2. Exit the survey" at Q1, the survey ends here.

------------------------- Page Break -------------------------

Q2.音声付き動画を見てロボットの声は何歳くらいの声に聞こえましたか？ 必須

【答えは１つです】

- ○ 1. 3歳児未満くらいの声
- ○ 2. 幼稚園児くらいの声
- ○ 3. 小学生くらいの声
- ○ 4. 中学生くらいの声
- ○ 5. 高校生くらいの声
- ○ 6. 大学生くらいの声
- ○ 7. 中年くらいの声
- ○ 8. 高齢者くらいの声
- ○ 9. 音声は聞こえなかった
- ○ 10. その他

【終了】Q2.で『9.音声は聞こえなかった』『10.その他』いずれかを選択した

---------- <次ページ> ----------

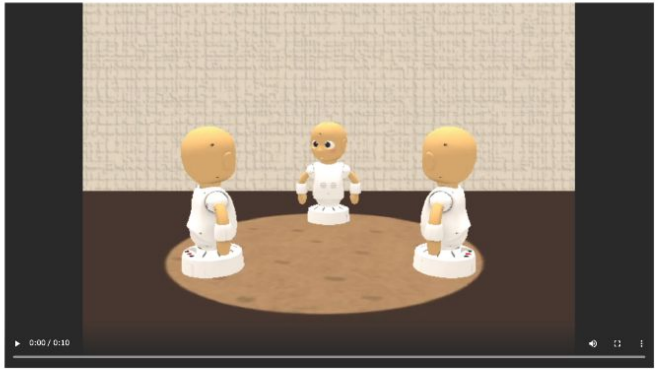

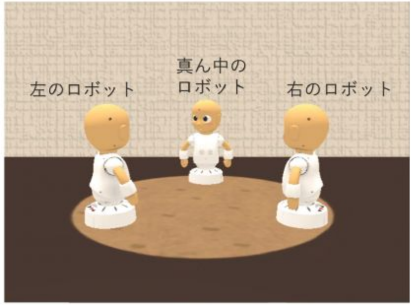

Q2. After watching the video with audio, how old did the robot's voice sound to you?

[Choose only one answer]

1. Younger than 3 years old
2. Like a kindergartener
3. Like an elementary school student
4. Like a junior high school student
5. Like a high school student
6. Like a university student
7. Like a middle-aged person
8. Like an elderly person
9. Couldn't hear the audio
10. Other

[End] If you selected "9. Couldn't hear the audio" or "10. Other" at Q2, the survey ends here.

------------------------- Page Break -------------------------

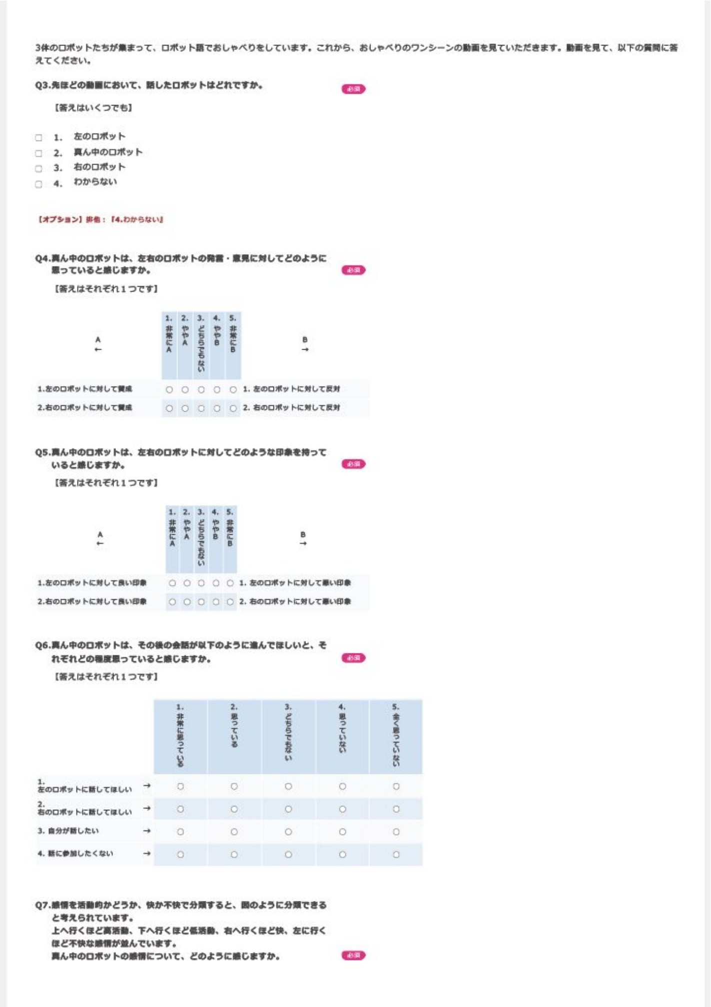

3体のロボットたちが集まって、ロボット語でおしゃべりをしています。これから、おしゃべりのワンシーンの動画を見ていただきます。動画を見て、以下の質問に答えてください。

Q3.先ほどの動画において、話したロボットはどれですか。 必須

【答えはいくつでも】

- ☐ 1. 左のロボット
- ☐ 2. 真ん中のロボット
- ☐ 3. 右のロボット
- ☐ 4. わからない

【オプション】排他：『4.わからない』

Q4.真ん中のロボットは、左右のロボットの発言・意見に対してどのように思っていると感じますか。 必須

【答えはそれぞれ１つです】

A ←	1. 非常にA	2. ややA	3. どちらでもない	4. ややB	5. 非常にB	B →
1.左のロボットに対して賛成	○	○	○	○	○	1. 左のロボットに対して反対
2.右のロボットに対して賛成	○	○	○	○	○	2. 右のロボットに対して反対

Q5.真ん中のロボットは、左右のロボットに対してどのような印象を持っていると感じますか。 必須

【答えはそれぞれ１つです】

A ←	1. 非常にA	2. ややA	3. どちらでもない	4. ややB	5. 非常にB	B →
1.左のロボットに対して良い印象	○	○	○	○	○	1. 左のロボットに対して悪い印象
2.右のロボットに対して良い印象	○	○	○	○	○	2. 右のロボットに対して悪い印象

Q6.真ん中のロボットは、その後の会話が以下のように進んでほしいと、それぞれどの程度思っていると感じますか。 必須

【答えはそれぞれ１つです】

		1. 非常に思っている	2. 思っている	3. どちらでもない	4. 思っていない	5. 全く思っていない
1. 左のロボットに話してほしい	→	○	○	○	○	○
2. 右のロボットに話してほしい	→	○	○	○	○	○
3. 自分が話したい	→	○	○	○	○	○
4. 話に参加したくない	→	○	○	○	○	○

Q7.感情を活動的かどうか、快か不快で分類すると、図のように分類できると考えられています。
上へ行くほど高活動、下へ行くほど低活動、右へ行くほど快、左に行くほど不快な感情が並んでいます。
真ん中のロボットの感情について、どのように感じますか。 必須

Three robots are gathered and chatting in robot language. You will now watch a scene from their conversation. Please watch the video and answer the following questions.

Q3. In the previous video, which robot(s) spoke?
[Select all that apply]
1. Left robot
2. Middle robot
3. Right robot
4. Don't know

[Option] Exclusive: "4. Don't know"

Q4. What do you think the middle robot felt about the comments/opinions of the left and right robots?
[Choose one for each]
A ←
1. Agree with the left robot
2. Agree with the right robot

B →
1. Oppose the left robot
2. Oppose the right robot

Scale: 1 (Strongly A) to 5 (Strongly B)

Q5. What kind of impression do you think the middle robot had toward the left and right robots?
[Choose one for each]
A ←
1. Good impression of the left robot
2. Good impression of the right robot

B →
1. Bad impression of the left robot
2. Bad impression of the right robot

Scale: 1 (Strongly A) to 5 (Strongly B)

Q6. To what extent do you think the middle robot wants the conversation to proceed in each of the following ways?
[Choose one for each]
1. Wants the left robot to speak →
2. Wants the right robot to speak →
3. Wants to speak themselves →
4. Does not want to participate in the conversation →

Scale: 1 (Strongly thinks so) to 5 (doesn’t strongly think so)

Q7. Emotions can be classified by how active and pleasant they are, as shown in the following diagram. Higher up = more active; lower = less active; right = more pleasant; left = more unpleasant. How do you feel about the emotion of the middle robot?

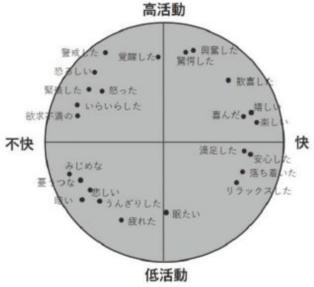

【答えはそれぞれ１つです】

A ←	1. 非常にA	2. ややA	3. どちらでもない	4. ややB	5. 非常にB	B →
1.低活動	○	○	○	○	○	1. 高活動
2.不快	○	○	○	○	○	2. 快

Q8.以下の項目について動画の真ん中のロボットの印象を評価してください。 必須

【答えはそれぞれ１つです】

A ←	1. 非常にA	2. ややA	3. どちらでもない	4. ややB	5. 非常にB	B →
1.死んでいる	○	○	○	○	○	1. 生きている
2.機械的な	○	○	○	○	○	2. 有機的な
3.人工的な	○	○	○	○	○	3. 生物的な
4.活気のない	○	○	○	○	○	4. 生き生きとした
5.不活発な	○	○	○	○	○	5. 対話的な
6.無関心な	○	○	○	○	○	6. 反応のある

【オプション】選択肢ランダマイズ

Q9.以下のそれぞれの項目について、この動画の真ん中のロボットはどのようであると感じますか。 必須

【答えはそれぞれ１つです】

		1. いつもそうだ	2. たいていそうだ	3. どちらともいえない	4. たいていそうでない	5. いつもそうでない
1. 他人と話していても、あまり会話が途切れないほうである	→	○	○	○	○	○
2. 他人にやってもらいたいことを、うまく指示することができる	→	○	○	○	○	○

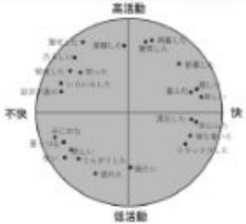

[Choose one for each axis]

A ←

1. Very A
2. Slightly A
3. Neutral
4. Slightly B
5. Very B

B →

1. Low activity
2. High activity
3. Unpleasant
4. Pleasant

Scale: 1 (Strongly A) to 5 (Strongly B)

Q8. Please evaluate the impression of the middle robot in the video on the following items.
[Choose one for each item]

A ←

1. Dead
2. Mechanical
3. Artificial
4. Lifeless
5. Inactive
6. Uninterested

B →

1. Alive
2. Organic
3. Biological
4. Lively
5. Interactive
6. Responsive

[Option] Randomize choices

Scale: 1 (Strongly A) to 5 (Strongly B)

Q9. For each of the following statements, how well do they describe the middle robot in the video?
[Choose one for each item]

1. Conversations don't often break down when talking with others
2. Can give clear instructions for what they want others to do

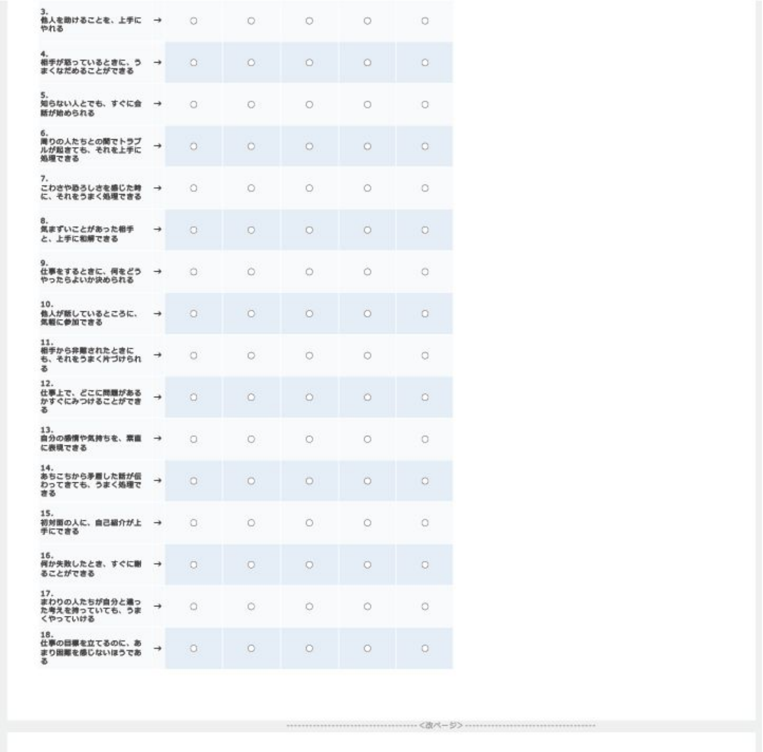

3. 他人を助けることを、上手にやれる	→	○	○	○	○	○
4. 相手が怒っているときに、うまくなだめることができる	→	○	○	○	○	○
5. 知らない人とでも、すぐに会話が始められる	→	○	○	○	○	○
6. 周りの人たちとの間でトラブルが起きても、それを上手に処理できる	→	○	○	○	○	○
7. こわさや恐ろしさを感じた時に、それをうまく処理できる	→	○	○	○	○	○
8. 気まずいことがあった相手と、上手に和解できる	→	○	○	○	○	○
9. 仕事をするときに、何をどうやったらよいか決められる	→	○	○	○	○	○
10. 他人が話しているところに、気軽に参加できる	→	○	○	○	○	○
11. 相手から非難されたときにも、それをうまく片づけられる	→	○	○	○	○	○
12. 仕事上で、どこに問題があるかすぐにみつけることができる	→	○	○	○	○	○
13. 自分の感情や気持ちを、素直に表現できる	→	○	○	○	○	○
14. あちこちから矛盾した話が伝わってきても、うまく処理できる	→	○	○	○	○	○
15. 初対面の人に、自己紹介が上手にできる	→	○	○	○	○	○
16. 何か失敗したとき、すぐに謝ることができる	→	○	○	○	○	○
17. まわりの人たちが自分と違った考えを持っていても、うまくやっていける	→	○	○	○	○	○
18. 仕事の目標を立てるのに、あまり困難を感じないほうである	→	○	○	○	○	○

-------------------- <改ページ> --------------------

1. Good at helping others
2. Can calm others down when they're angry
3. Can start conversations easily even with strangers
4. Can manage conflicts well with those around them
5. Can handle fear or scary situations well
6. Can reconcile smoothly with someone after an awkward moment
7. Can decide what and how to do tasks at work
8. Can join ongoing conversations comfortably
9. Can resolve criticism from others effectively
10. Can identify problems quickly in work situations
11. Can express emotions and feelings honestly
12. Can deal well with contradictory stories from various sources
13. Can introduce themselves well to new people
14. Can apologize quickly when making a mistake
15. Can get along with people who have different views
16. Finds it easy to set work goals

Scale: 1 (Strongly A) to 5 (Strongly B)

------------------------- Page Break -------------------------

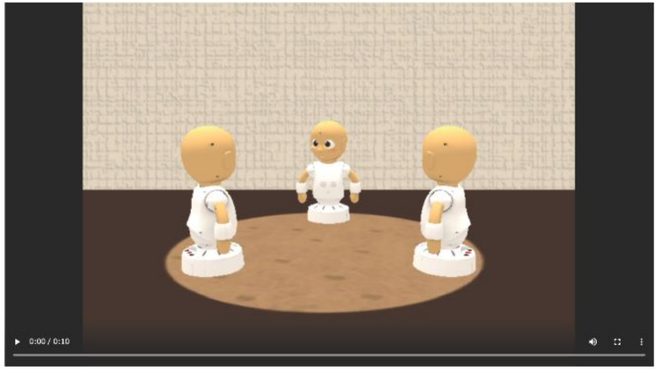

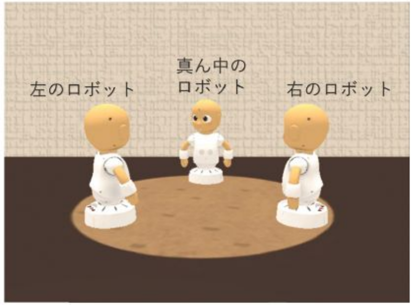

3体のロボットたちが集まって、ロボット語でおしゃべりをしています。これから、おしゃべりのワンシーンの動画を見ていただきます。動画を見て、以下の質問に答えてください。

Q10.先ほどの動画において、話したロボットと移動したロボットはどれですか。 必須

【答えはそれぞれいくつでも】

	1.左のロボット	2.真ん中のロボット	3.右のロボット	4.どのロボットでもない
1. 話したロボット →	☐	☐	☐	☐
2. 移動したロボット →	☐	☐	☐	☐

【オプション】排他：『4.どのロボットでもない』『5.わからない』

Q11.真ん中のロボットは、左右のロボットの発言・意見に対してどのように思っていると感じますか。 必須

【答えはそれぞれ１つです】

Three robots are gathered and chatting in robot language. You will now watch a scene from their conversation. Please watch the video and answer the following questions.

Q10. In the previous video, which robots spoke and which moved?

[Select all that apply for each]

1. Robots that spoke →
2. Robots that moved →

1. Left robot
2. Middle robot
3. Right robot
4. None of the robots
5. Don't know

[Option] Exclusive: "4. None of the robots", "5. Don't know"

Q11. What do you think the middle robot felt about the comments/opinions of the left and right robots?

[Choose one for each]

A ←	1. 非常にA	2. ややA	3. どちらでもない	4. ややB	5. 非常にB	B →
1.左のロボットに対して賛成	○	○	○	○	○	1. 左のロボットに対して反対
2.右のロボットに対して賛成	○	○	○	○	○	2. 右のロボットに対して反対

Q12.真ん中のロボットは、左右のロボットに対してどのような印象を持っていると感じますか。 必須

【答えはそれぞれ１つです】

A ←	1. 非常にA	2. ややA	3. どちらでもない	4. ややB	5. 非常にB	B →
1.左のロボットに対して良い印象	○	○	○	○	○	1. 左のロボットに対して悪い印象
2.右のロボットに対して良い印象	○	○	○	○	○	2. 右のロボットに対して悪い印象

Q13.真ん中のロボットは、その後の会話が以下のように進んでほしいと、それぞれどの程度思っていると感じますか。 必須

【答えはそれぞれ１つです】

		1. 非常に思っている	2. 思っている	3. どちらでもない	4. 思っていない	5. 全く思っていない
1. 左のロボットに話してほしい	→	○	○	○	○	○
2. 右のロボットに話してほしい	→	○	○	○	○	○
3. 自分が話したい	→	○	○	○	○	○
4. 話に参加したくない	→	○	○	○	○	○

Q14.感情を活動的かどうか、快か不快で分類すると、図のように分類できると考えられています。
上へ行くほど高活動、下へ行くほど低活動、右へ行くほど快、左に行くほど不快な感情が並んでいます。
真ん中のロボットの感情について、どのように感じますか。 必須

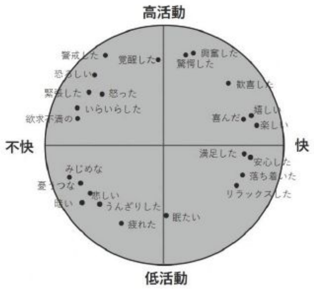

A ←
1. Agree with the left robot
2. Agree with the right robot

B →
1. Oppose the left robot
2. Oppose the right robot

Scale: 1 (Strongly A) to 5 (Strongly B)

Q12. What kind of impression do you think the middle robot had toward the left and right robots?
[Choose one for each]

A ←
1. Good impression of the left robot
2. Good impression of the right robot

B →
1. Bad impression of the left robot
2. Bad impression of the right robot

Scale: 1 (Strongly A) to 5 (Strongly B)

Q13. To what extent do you think the middle robot wants the conversation to proceed in each of the following ways?
[Choose one for each]
1. Wants the left robot to speak →
2. Wants the right robot to speak →
3. Wants to speak themselves →
4. Does not want to participate in the conversation →

Scale: 1 (Strongly A) to 5 (Strongly B)

Q14. Emotions can be classified by how active and pleasant they are, as shown in the diagram. Higher up = more active; lower = less active; right = more pleasant; left = more unpleasant. How do you feel about the emotion of the middle robot?

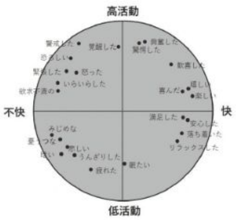

【答えはそれぞれ１つです】

A ←	1.非常にA	2.ややA	3.どちらでもない	4.ややB	5.非常にB	B →
1.低活動	○	○	○	○	○	1. 高活動
2.不快	○	○	○	○	○	2. 快

Q15.以下の項目について動画の真ん中のロボットの印象を評価してください。 必須

【答えはそれぞれ１つです】

A ←	1. 非常にA	2. ややA	3. どちらでもない	4. ややB	5. 非常にB	B →
1.不活発な	○	○	○	○	○	1. 対話的な
2.人工的な	○	○	○	○	○	2. 生物的な
3.無関心な	○	○	○	○	○	3. 反応のある
4.機械的な	○	○	○	○	○	4. 有機的な
5.死んでいる	○	○	○	○	○	5. 生きている
6.活気のない	○	○	○	○	○	6. 生き生きとした

【オプション】選択肢ランダマイズ

Q16.以下のそれぞれの項目について、この動画の真ん中のロボットはどのようであると感じますか。 必須

【答えはそれぞれ１つです】

		1. いつもそうだ	2. たいていそうだ	3. どちらともいえない	4. たいていそうでない	5. いつもそうでない
1. 他人と話していても、あまり会話が途切れないほうである	→	○	○	○	○	○
2. 他人にやってもらいたいことを、うまく指示することができる	→	○	○	○	○	○
3. 他人を助けることを、上手にやれる	→	○	○	○	○	○
4. 相手が怒っているときに、うまくなだめることができる	→	○	○	○	○	○
5. 知らない人とでも、すぐに会話が始められる	→	○	○	○	○	○
6. 周りの人たちとの間でトラブルが起きても、それを上手に処理できる	→	○	○	○	○	○
7. こわさや恐ろしさを感じた時に、それをうまく処理できる	→	○	○	○	○	○
8. 気まずいことがあった相手と、上手に和解できる	→	○	○	○	○	○
9. 仕事をするときに、何をどうやったらよいか決められる	→	○	○	○	○	○
10. 他人が話しているところに、気軽に参加できる	→	○	○	○	○	○
11. 相手から非難されたときにも、それをうまく片づけられる	→	○	○	○	○	○
		○	○	○	○	○

[Choose one for each axis]

1. A: Low activity / B: High activity
2. A: Unpleasant / B: Pleasant

Scale: 1 (Strongly A) to 5 (Strongly B)

Q15. Please evaluate the impression of the middle robot in the video on the following items.
[Choose one for each item]
A ←
1. Inert
2. Artificial
3. Apathetic
4. Mechanical
5. Dead
6. Stagnant

B →
1. Interactive
2. Lifelike
3. Responsive
4. Organic
5. Alive
6. Lively

[Option] Randomize choice
Scale: 1 (Strongly A) to 5 (Strongly B)

Q16. For each of the following statements, how well do they describe the middle robot in the video?

[Choose one for each item]
(Repeat of Q9 item list)
Scale: 1 (Strongly so) to 5 (not strongly so)
1. When talking with others, it tends not to have many interruptions in the conversation.
2. It can effectively instruct others on what it wants them to do.
3. It can skillfully help others.
4. When someone is angry, it can calm them down well.
5. It can quickly start a conversation even with strangers.
6. Even if trouble arises with people around it, it can handle the situation skillfully.
7. When it feels scared or terrified, it can manage those feelings well.
8. It can skillfully reconcile with someone with whom there has been an awkward situation.
9. When working, it can decide what needs to be done and how to do it.
10. It can casually join in when others are talking.
11. Even when criticized by others, it can handle it well.
12. In its work, it can quickly find where problems lie.
13. It can frankly express its own emotions and feelings.
14. Even when receiving contradictory information from various sources, it can handle it well.
15. It can skillfully introduce itself to people it meets for the first time.
16. When it makes a mistake, it can apologize promptly.
17. Even if the people around it have different ideas from its own, it can get along well with them.
18. It doesn't find it very difficult to set work goals.

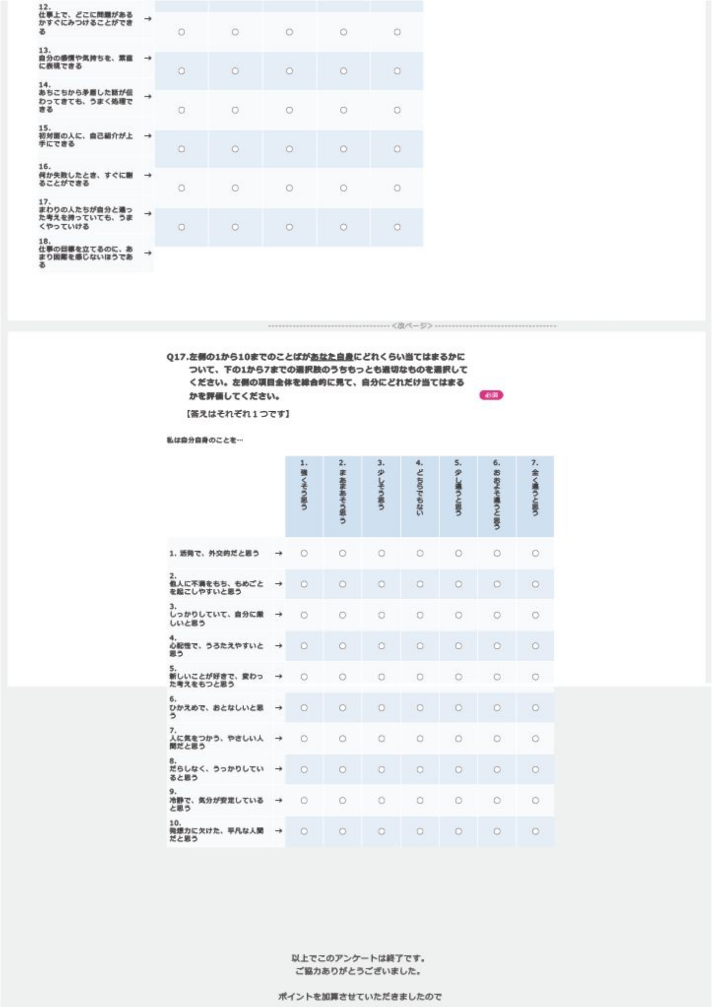

12. 仕事上で、どこに問題があるかすぐにみつけることができる →	○	○	○	○	○
13. 自分の感情や気持ちを、素直に表現できる →	○	○	○	○	○
14. あちこちから矛盾した話が伝わってきても、うまく処理できる →	○	○	○	○	○
15. 初対面の人に、自己紹介が上手にできる →	○	○	○	○	○
16. 何か失敗したとき、すぐに謝ることができる →	○	○	○	○	○
17. まわりの人たちが自分と違った考えを持っていても、うまくやっていける →	○	○	○	○	○
18. 仕事の目標を立てるのに、あまり困難を感じないほうである →					

<改ページ>

Q17.左側の1から10までのことばがあなた自身にどれくらい当てはまるかについて、下の1から7までの選択肢のうちもっとも適切なものを選択してください。左側の項目全体を総合的に見て、自分にどれだけ当てはまるかを評価してください。 必須

【答えはそれぞれ１つです】

私は自分自身のことを…

	1. 強くそう思う	2. まあまあそう思う	3. 少しそう思う	4. どちらでもない	5. 少し違うと思う	6. おおよそ違うと思う	7. 全く違うと思う
1. 活発で、外交的だと思う →	○	○	○	○	○	○	○
2. 他人に不満をもち、もめごとを起こしやすいと思う →	○	○	○	○	○	○	○
3. しっかりしていて、自分に厳しいと思う →	○	○	○	○	○	○	○
4. 心配性で、うろたえやすいと思う →	○	○	○	○	○	○	○
5. 新しいことが好きで、変わった考えをもつと思う →	○	○	○	○	○	○	○
6. ひかえめで、おとなしいと思う →	○	○	○	○	○	○	○
7. 人に気をつかう、やさしい人間だと思う →	○	○	○	○	○	○	○
8. だらしなく、うっかりしていると思う →	○	○	○	○	○	○	○
9. 冷静で、気分が安定していると思う →	○	○	○	○	○	○	○
10. 発想力に欠けた、平凡な人間だと思う →	○	○	○	○	○	○	○

以上でこのアンケートは終了です。
ご協力ありがとうございました。

ポイントを加算させていただきましたので

Q17. Regarding the 10 statements on the left, please select the most appropriate option from 1 to 7 that describes how well each applies to you.
[Choose one for each statement]
Statements:

1. I think I am active and outgoing
2. I tend to have complaints about others and often cause conflicts
3. I am diligent and strict with myself
4. I am anxious and easily flustered
5. I like new things and have unconventional ideas
6. I am reserved and quiet
7. I am considerate and kind
8. I am sloppy and careless
9. I am calm and emotionally stable
10. I lack creativity and am an ordinary person

Scale:

1. Strongly agree
2. Somewhat agree
3. Slightly agree
4. Neither agree nor disagree
5. Slightly disagree
6. Somewhat disagree
7. Strongly disagree

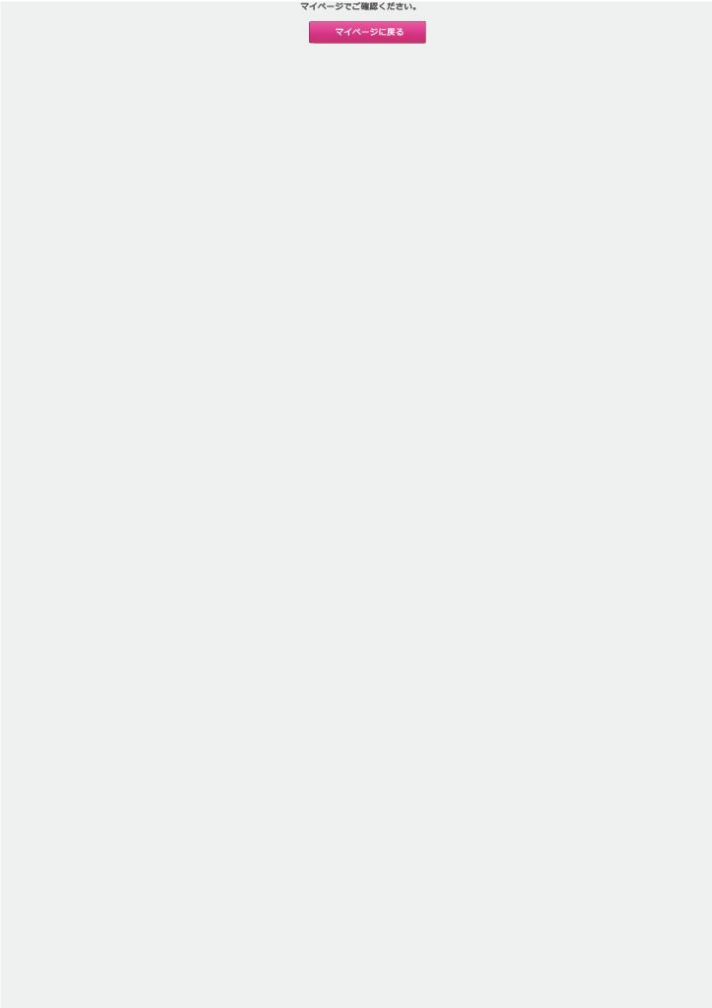

This concludes the survey. Thank you for your cooperation. Points have been added. Please check your My Page.

Return to My Page
